# Aux-MVNet: Auxiliary Classifier-Based Multi-View Convolutional Neural Network for Maxillary Sinusitis Diagnosis on Paranasal Sinuses View

**DOI:** 10.3390/diagnostics12030736

**Published:** 2022-03-18

**Authors:** Sang-Heon Lim, Jong Hoon Kim, Young Jae Kim, Min Young Cho, Jin Uk Jung, Ryun Ha, Joo Hyun Jung, Seon Tae Kim, Kwang Gi Kim

**Affiliations:** 1 Department of Health Sciences and Technology, Gachon Advanced Institute for Health Sciences and Technology, Seongnam-si 21565, Korea; smion123@gachon.ac.kr; 2 Department of Biomedical Engineering, College of Health Science, Gachon University, Incheon 21565, Korea; jhkim2020@gachon.ac.kr (J.H.K.); youngjae@gachon.ac.kr (Y.J.K.); 3 Department of Otolaryngology-Head & Neck Surgery, Gill Medical Center, College of Medicine, Incheon 21565, Korea; whatupcho@gilhospital.com (M.Y.C.); jeong3367@gilhospital.com (J.U.J.); bewitch@gilhospital.com (J.H.J.); 4 Department of Otolaryngology-Head & Neck Surgery, Armed Forces Capital Hospital, Seongnam-si 21565, Korea; scherzojj@naver.com

**Keywords:** paranasal sinus view, sinusitis, artificial intelligence, CNN, multi-view network

## Abstract

Computed tomography (CT) is undoubtedly the most reliable and the only method for accurate diagnosis of sinusitis, while X-ray has long been used as the first imaging technique for early detection of sinusitis symptoms. More importantly, radiography plays a key role in determining whether or not a CT examination should be performed for further evaluation. In order to simplify the diagnostic process of paranasal sinus view and moreover to avoid the use of CT scans which have disadvantages such as high radiation dose, high cost, and high time consumption, this paper proposed a multi-view CNN able to faithfully estimate the severity of sinusitis. In this study, a multi-view convolutional neural network (CNN) is proposed which is able to accurately estimate the severity of sinusitis by analyzing only radiographs consisting of Waters’ view and Caldwell’s view without the aid of CT scans. The proposed network is designed as a cascaded architecture, and can simultaneously provide decisions for maxillary sinus localization and sinusitis classification. We obtained an average area under the curve (AUC) of 0.722 for maxillary sinusitis classification, and an AUC of 0.750 and 0.700 for the left and right maxillary sinusitis, respectively, using the proposed network.

## 1. Introduction

Rhinosinusitis is defined as inflammation of the nasal cavity and paranasal sinuses (PNS). The prevalence of chronic rhinosinusitis in the general population based on sinus radiology and symptoms ranges from 3.0% to 6.4% of clinically substantiated chronic rhinosinusitis (CRS) in a randomly selected group of subjects [1]. While the diagnosis of acute rhinosinusitis is based on history and physical examination, chronic rhinosinusitis and recurrent acute rhinosinusitis are diagnosed by symptoms and the presence of disease on either a sinus examination CT scan and/or endoscopy [2]. Imaging findings do not always correlate with symptoms. Therefore, imaging should confirm the presenting signs and symptoms [3]. Radiographs can detect mucosal thickening, air fluid levels, opacities of the paranasal sinuses, anatomic variants, and foreign bodies [4]. The diagnostic sensitivity of the paranasal sinus view is very low due to the opacification of the bone by overlapping with some anatomical structures [5]. Waters’ view has its limitations in the diagnosis of sinusitis of the maxillary sinuses and its contribution to the diagnosis of lesions in the other maxillary sinuses is very poor [6]. Therefore, CT of the paranasal sinuses has become the gold standard for sinus imaging in complicated sinus disease [4]. However, PNS view is usually examined at their first visit in patients who visit the ENT (ear, nose and throat) department for nasal symptoms of sinusitis.

Meanwhile, deep learning techniques are highly appreciated as a tool for a variety of problems in the field of medical image analysis and computer-aided diagnosis (CAD) [7,8,9,10,11,12]. In fact, several deep learning-based studies have reported invaluable results related to the diagnosis of sinusitis in the PNS view. One of the related studies has shown that deep learning-based diagnosis of maxillary sinusitis on Waters’ radiograph can achieve a better area under the receiver operating characteristic curve (AUC) than conventional methods, and also has comparable sensitivity and specificity to that of the radiologist [13]. Alternatively, based on the fact that the diagnosis of maxillary sinusitis in the ENT department is usually made using both Caldwell’s and Waters’ view, a multi-view model analyzing the two different views simultaneously was proposed and showed higher AUC than the previous study [13] which used Waters’ view for the diagnosis of frontal, ethmoid and maxillary sinusitis only. Another noteworthy point of the approach is the construction of a cascaded network by introducing a detector network at the preceding stage of the classification network, so that the method could bypass the time-consuming and laborious task of manually specifying the sinus region [14]. Although the study obviously showed better performance than conventional sinusitis diagnosis, which relies solely on human decisions [1], there are still critical issues that need to be addressed. First, the findings obtained in the study did not distinguish between the left and right sides of the PNS view. However, to assist medical personnel in their clinical decisions, it is essential that a CAD tool indicates the correct side of the sinus: the left or right side. More importantly, the related work aimed to classify three groups, i.e., the severity of normal-healthy, sinusitis (inflammation over 4 mm) and air-fluid, but this problem is unlikely to be an urgent issue in a real clinical field. In fact, the problem of distinguishing a healthy sinus from a case with severe sinusitis should be less challenging for both computer vision and human specialists.

Inspired by the achievements and limitations of the aforementioned studies, this study proposes a CNN-based multi-view model capable of accurately diagnosing maxillary sinusitis using Waters’ and Caldwell’s views. To accurately diagnose sinusitis, the maxillary sinus was reckoned as a region of interest (ROI) using a region proposal network (RPN), which was eventually used to diagnose paranasal sinusitis. We attempted to find the most suitable deep learning network for diagnosing maxillary sinusitis among six CNN-based multi-view networks, based on the combination of three different CNN models. Our proposed model was able to accurately locate the paranasal sinuses in the PNS view and determine the development of paranasal sinusitis, making it possible to create an objective and reliable CAD system without the help of CT.

## 2. Materials and Methods

### 2.1. Study Populations

This retrospective study collected radiographic PNS series from 1491 patients evaluated for paranasal sinusitis at Gachon University Gil medical center between 2007 and 2020. To account for pneumatization of the paranasal sinuses, data from patients under 19 years. In addition, those patients with air-fluid were excluded via clinical decision of otolaryngologist, and finally only 587 PNS series from patients (279 males and 308 females) aged 20 to 90 years were examined. Of the 587 patients, 446 were patients with maxillary sinus, and 141 were normal healthy patients.

### 2.2. Radiograph Acquisition

X-ray image acquisition was done under the following conditions. Tube voltage ranged from 63 to 85 kVp, with a mean value of 73.35, and tube-current covered 195–800 mA with a mean value of 391.64 mA. To ensure an optimal contrast, the window center and window width were set to 0–255 in the range of 1788 to 14,162; and 2075 to 16,647, respectively; the mean values were 5031.49 and 8963.16, respectively.

### 2.3. Labeling

The CT scan of the maxillar served as the ground truth (gold standard) of sinusitis for deciding paranasal sinusitis during model learning. Paranasal sinusitis was labelled separately for right and left maxillary sinus, which was determined according to the following scoring criteria: level-0 (healthy) if the proportion of inflammation in the right or left maxillary was less than 2 mm, level-1 in the case of 2–5 mm, and lastly, level-3 if greater than 5 mm. This labelling process was applied consistently to both datasets collected from Waters’ and Caldwell’s views. The left and right maxillary sinuses are indistinguishable in the lateral view, so Waters’ view and Caldwell’s view were used in this study except for the lateral view. From our dataset, patients with sinusitis level-1 on the left sinus were 83, and 180 patients were level-2. In the right sinus, patients with sinusitis level-1 were 74, and 193 patients were level-2. We included 324 normal healthy left maxillary sinus participants and 320 normal healthy right maxillary sinus participants. Two well-trained otorhinolaryngologists labeled all radiographs: ENT professors with 10 and 20 years of experience.

### 2.4. Experimental Design

In this study, the deep learning models were evaluated with a five-fold cross-validation method, in which the problem of dataset imbalance was mitigated by evenly distributing the ratio of patients with paranasal sinusitis and normal-healthy; each fold consists of approximately 80% of patients with sinusitis and 20% normal-healthy.

### 2.5. Region of Sinus Detection

For the detection of the maxillary, we used RPN based on a feature pyramid network using Resnet50 as the backbone also known as RetinaNet [15,16]; the source code is available at https://github.com/fizyr/keras-retinanet (accessed on 10 November 2021). During RPN learning, the right and left side maxillary sinus were trained separately as individual objects.

Prior to the implementation of the algorithm, i.e., learning, validation, and testing, all input data were normalized to fix the intensity to a uniform range of −1 to 1, and resized to a resolution of 512 × 512. To facilitate the learning process, we introduced a transfer-learning scheme where the parameters are initialized with ImageNet pre-trained weight [17]. The RetinaNet outputs the bounding box coordinates for the sinus region and the probability value (0–1) for the left or right class for the corresponding region.

### 2.6. Sinusitis Classification

**Figure 1:**
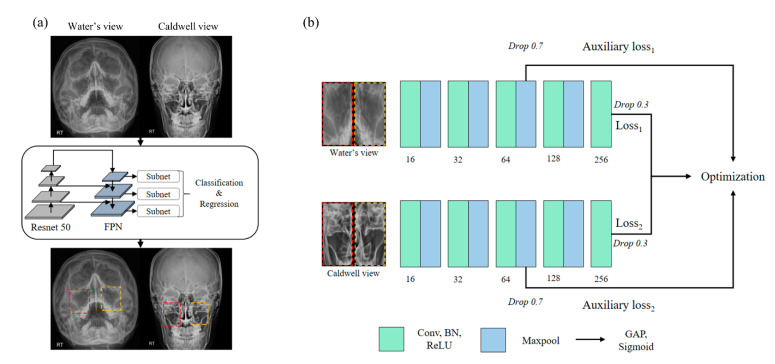
(**a**) Displays the overview of the framework to detect paranasal sinusitis using RetinaNet. Red and orange bounding boxes indicate the region of left and right sinuses, respectively. (**b**) provides the schematic diagram of Aux-MVNet that represents the roles of the individual blocks. Abbreviations: FPN, feature pyramid network; Drop, drop out; BN, batch normalization; ReLU, rectified linear unit; GAP, global average pooling.

This study is based on the concept of ablation study [18] where three internal modules, i.e., basic CNN blocks, dense blocks [19], and inception blocks [20] were tested by inserting them individually into the multi-view network (MVNet) shown in Figure 1. The CNN model used in this study consists of four resolution steps and uses batch normalization and rectified linear unit (ReLU). We modified the open-access code of the multi-view CNN [21] to make it usable for our dataset; the original code is available at https://github.com/suhangpro/mvcnn (accessed on 10 November 2021). The X-ray images from Waters’ view and Caldwell’s view were independently encoded in the network, while two different views were used simultaneously for the network optimization, as shown in Figure 1. In contrast, to stabilize the optimization, we introduced an auxiliary classifier [22] with global average pooling (GAP) and sigmoid activation function in the third resolution step. The auxiliary losses were only used for network optimization and not for sinusitis inference. In addition, a drop out scheme was applied to the third convolution block and the last layer resulting in an output of 70% and 30%, respectively.

Three CNN models were derived into six CNN models by adding an auxiliary loss function in the layer corresponding to the third resolution step of each model. Table 1 compares the internal structures of the four CNN-based models tested in this work, each of which yields an additional version by hiring the layers with the symbol *; in total, we tested six models.

The networks tested consist of two individual networks for Waters’ and Caldwell’s views, and the two individual networks compute the probability of six classes from Waters’ and Caldwell’s views, respectively, i.e., the probabilities of normal healthy, sinusitis level-1 and level-2 with respect to either the left or right sinus. The final output (Ŷ) of the multi-view network is determined by the following formula:

(1) $$Ŷ=\frac{P_{w}\times 0.6+P_{c}\times 0.4}{2}$$

where $P_{w}$ and $P_{c}$ denote the probabilities computed from networks taking Waters’ and Caldwell’s views as the input.

### 2.7. Implementation Details

We used Keras version 2.2.5 (TensorFlow-GPU backend, version 1.15.4) for deep learning analysis and Simple-ITK (ver. 1.2.0) for radiograph preprocessing on Python version 3.6.12. This study was conducted on a ppc64le central processing unit (CPU) architecture of the IBM POWER9 system and NVIDIA Tesla V100 graphics-processing unit (GPU).

The Adam optimizer [23] (initial learning-rate = 0.001) and the binary cross-entropy loss function were used for each network. We also used the sigmoid activation function to output the network’s inference results. The hyper-parameters used for our model are as follows: batch-size = 32; total training epochs = 500; learning-rate reduction factor = 0.1 and learning-rate reduction patience = 10. Lastly, the early stopping approach was applied with 50 patience.

All networks were designed to perform the multi label classification based on the comparison of probabilities resulting from 6 sigmoid functions corresponding to 6 classes, generated by the combination of 3 sinusitis levels and two sides, the left and right sinus regions.

## 3. Results

### 3.1. Region of Sinus Detection

**Figure 2:**
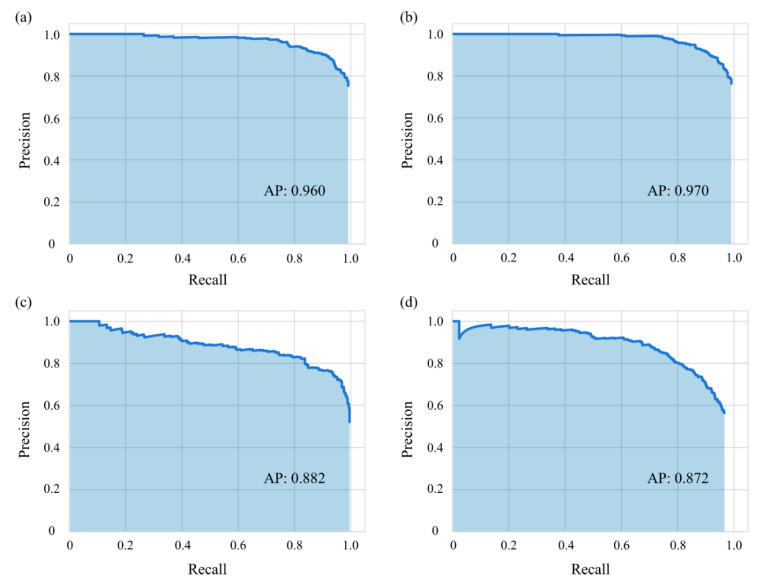
(**a**,**b**) show p-r curves demonstrating the detection performances of left and right maxillaries in Waters’ view, and (**c**,**d**) correspond to those of left and right in Caldwell’s view. Abbreviation: AP, average precision.

Prior to the diagnosis of paranasal sinusitis, the detection process for left and right maxillaries was performed. Figure 2 shows precision-recall (p-r) curves representing the performance of RetinaNet in detecting left and right maxillaries from Waters’ and Caldwell’s views. In this work, the average precision (AP) was used as a metric to evaluate the detection performance with respect to maxillary. If the intersection over union (IOU) value was above 0.5, it was considered to be a true positive [24,25].

We obtained 0.960 and 0.970 of AP for the left and right sinus detection task via RetinaNet on Waters’ view, and AP values for the left and right sinus detection on Caldwell’s view were 0.882 and 0.872. In terms of IOU scores, from Waters’ view we obtained 0.797 ± 0.096 and 0.789 ± 0.097 of average IOU in left and right and from Caldwell’s view, the average IOU values were 0.724 ± 0.125 and 0.720 ± 0.131 in left and right, respectively.

**Figure 3:**
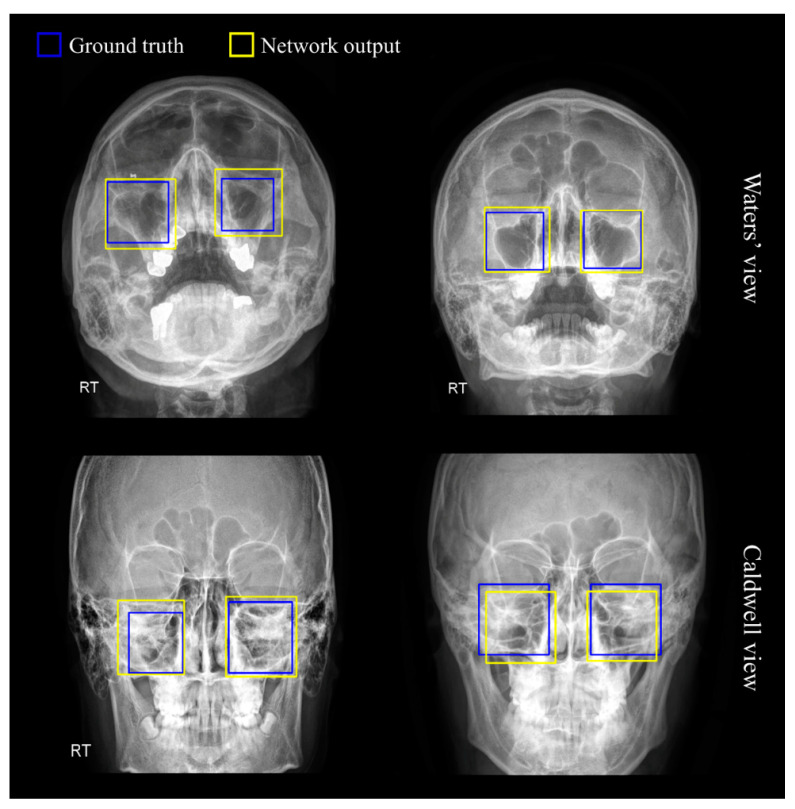
Representative examples of maxillary sinus detection results via RetinaNet. The blue bounding boxes indicate the ground truth and the yellow boxes indicate the prediction results of the neural network.

Figure 3 illustrates the sinus detection results obtained by RetinaNet, where the blue boxes refer to the gold-standard of maxillary regions, and the yellow boxes indicate the prediction results. As observed in the figures, the detection results of sinus were satisfactory in both views; the degree of discrepancies between the two boxes can be considered negligible. Multiple bounding box coordinates were recommended by the RPN as possible candidates for the left or right sinuses, and the sinus region with the highest probability was finally selected and cropped. 

### 3.2. Sinusitis Classification

To compare the discriminative performance between the presented classification models, we used the average AUC which can be a measure for a comprehensive evaluation of a classification problem. The outputs predicted from each model were labeled with true or false with respect to three classes, i.e., levels 0, 1, and 2, which were used to analyze the receiver operating characteristics (ROC) individually for the different classes. Table 2 compares the micro average AUC values of six classification networks created with the concept of an ablation study depending on the application of the auxiliary approach to three different multi-view networks. 

As for the classification performance for the models without applying the auxiliary classifier, the network applying the dense module outperformed its counterparts with an average AUC of 0.706. When comparing all candidate models to justify the effectiveness of the auxiliary classifier, the basic MVNet hiring auxiliary classifier (Aux-MVNet) showed the best performance with an average AUC of 0.722. The *p*-values in Table 2 numerically support the significance of the comparison results. For the classification performance, we used the ROC comparison approach. As shown in the numerical results, the auxiliary classifier obviously helped to improve the performance.

Looking at the evaluation results for the right maxillary sinusitis dataset only, the Aux-MVNet with the inception modules showed the highest AUC value. In the overall results considering both sides (left and right), the Aux-MVNet outperformed the network showing a much higher AUC score. Overall, the evaluation results of this experiment revealed that Aux-MVNet is the most likely to classify the degree of maxillary sinusitis among the deep learning architectures developed in this work. Regarding the effectiveness of the introduction of the auxiliary classifier, it led to a significant improvement in the AUC for all networks used, although the degree of contribution varies according to the models tested.

**Figure 4:**
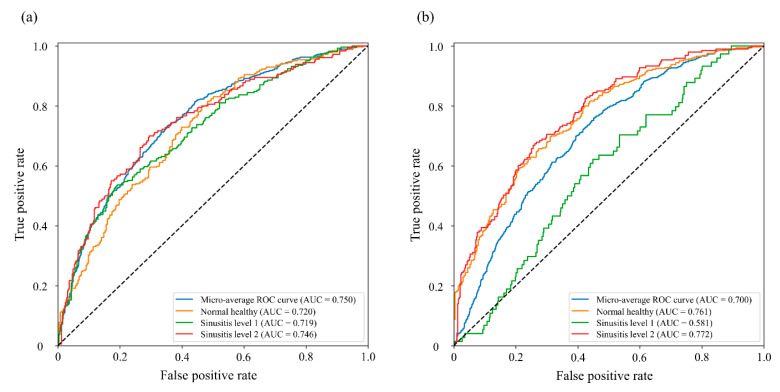
The graphs compare the ROC curves in relation to each class which are divided based on the severity of sinusitis, and the area below the curve indicates the AUC. The graphs were generated by MVNet with Auxiliary loss schemes. The AUC and ROC analyses for the left side (**a**) and right side (**b**) of sinusitis classification performance. The blue, orange, green and red lines indicate the evaluation for total classes (micro-average ROC), normal healthy, level-1, and level-2, respectively. Abbreviations: ROC, receiver operating curve; AUC, area under the curve.

The graphs of Figure 4 show in detail the performance of the Aux-MVNet which was found to be the best-fitting model in Table 2. The AUC scores of the graphs corresponding to three classes, i.e., normal, level-1, level-2 were 0.740, 0.639 and 0.759, respectively, in the average AUC of left and right sinusitis; we evaluated our network for left and right sinusitis. The AUC score of most classes was above 0.700, but in the right sinusitis level-1 the AUC was 0.581.

In terms of the discriminative ability of Aux-MVNet for the severity of the disease, i.e., normal healthy, sinusitis level-1 and level-2, the average sensitivity and specificity in left sinus were 0.677 ± 0.107 and 0.683 ± 0.112, respectively, and in the right sinus were 0.666 ± 0.032 of sensitivity and 0.658 ± 0.077 of specificity. Moreover, we obtained an average accuracy of 0.689, 0.654 and 0.659 for the left, right and total sinus regions, respectively. The corresponding diagnostic performance of the Aux-MVNet was evaluated separately for left and right sinusitis, with the optimal association criterion determined by Youden’s J statistic [26,27] (Table 3). All corresponding ROC curves for each class resulted in a statistically significant difference from the identity line that was significantly below 0.05 of the *p*-value.

We used MedCalc Statistical Software (ver. 14.8.1, https://www.medcalc.org (accessed on 10 November 2021)) and Scikit-learn (ver. 0.22.1, https://scikit-learn.org (accessed on 10 November 2021)) for network assessment.

To investigate the effectiveness of introducing the auxiliary classifier, we compared the activation images revealed by the gradient-weighted class activation mapping (Grad-CAM) [28,29] method with the outputs from the third layer convolutional block in Aux-MVNet and MVNet without the auxiliary classifier; the auxiliary classifier is positioned in the third convolutional block in the network.

**Figure 5:**
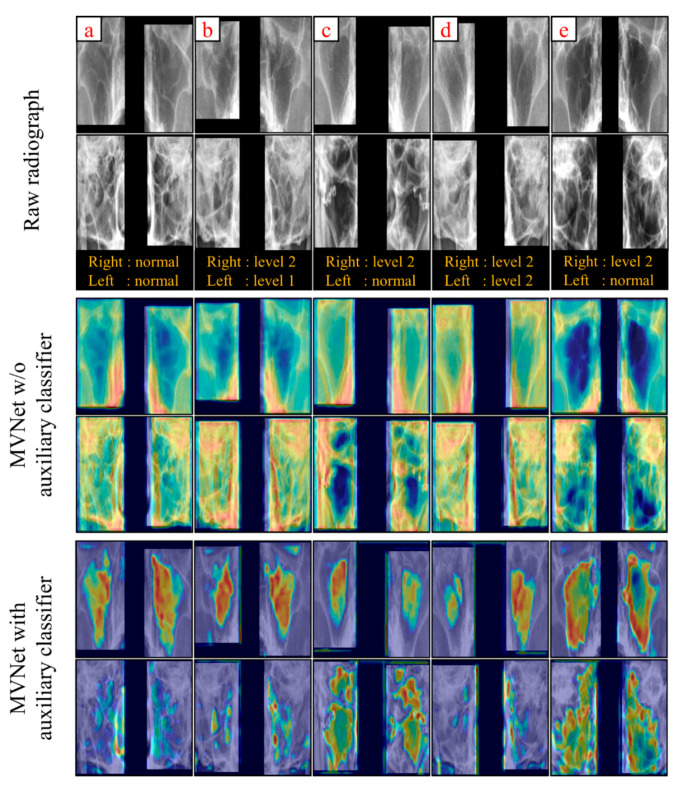
Grad-CAM analysis from the images of 5 subjects in the 3rd convolution block of MVNet and Aux-MVNet (**a**–**e**). The Gold-standard of sinusitis levels were presented below the raw images. Odd rows represent Waters’ view, and even rows represent Caldwell’s view.

As shown in Figure 5, it was ensured that the network used the auxiliary classifier that enables it to more effectively activate the areas corresponding to the maxillary sinus while the MVNet without auxiliary classifier tends to focus on more bony areas that do not interest us. The corresponding results show that the auxiliary classifier method can train the MVNet efficiently.

## 4. Discussion

In this work, we developed an auxiliary classifier-based multi-view network, Aux-MVNet, for classifying sinusitis in PNS view. The proposed network showed better discriminative power for sinusitis than other candidates developed with the same intention. More specifically, our network perfectly distinguished the entirety of sinusitis levels, with an average AUC score on the left and right sides of the sinus that was greater than 0.720. Furthermore, we used Grad-CAM to visually reveal the effectiveness of the auxiliary classifier and analyzed the Grad-CAM output of the third convolutional layer. Examination of the resulting activated images clearly confirmed that the network with the auxiliary classifier was focused on the sinus regions. In contrast, the network without the introduction of the auxiliary classifier, only the bone regions were considered. 

Compared with the performance of recent studies [13,14] that reported an AUC of 0.93 and 0.88 for sinusitis classification tasks over a deep neural network, our work showed a relatively low AUC. However, it should be noted that there are several differences in the research setting from the previous works. First, the labels for statistical analysis are dichotomized as normal or sinusitis while in our work each statistical analysis was performed for each of the three classes. Moreover, our labeling strategy for maxillary sinusitis was constructed to solve the difficult problems faced by ENT specialists. In the previous study, level-1 was used for maxillary sinusitis of 4 mm or more, level-2 for air-fluid, and level-3 for total opacification while we designed class level-1 to be 2~5 mm and level-2 to 5 mm or more. In other words, we divided the classes for maxillary sinusitis thickness into more finely-grained levels, and specifically excluded air-fluid which can easily be identified by the conventional technique.

To achieve the best possible results, we had to focus primarily on finding a solver that solves the inherent problem that makes the straightforward analysis of radiograph images difficult. Paranasal sinus images taken using X-rays are likely to be shadowed by the skull and various anatomical structures making it a difficult task to detect paranasal sinusitis and to extract a salient feature map from X-ray images as opposed to CT. In cases where the raw data information is scarce and unclear, the introduction of relatively shallow networks is usually known to be an effective method to prevent overfitting and optimize the models. However, the approach of simply reducing the depth of the network has obvious limitations in improving predictive ability; a shallow model may not capture important features of the image. Accordingly, we sought to improve the model performance by adding an auxiliary classifier rather than massively simplifying the model’s network.

Considering the characteristics of the dataset determined by the environment described above, we postulated that deep supervised learning where auxiliary loss is introduced into a relatively shallow network would be a better strategy for reliable classification of sinusitis [30,31], rather than designing a deep network model by concatenating possible modules, i.e., dense or inception blocks. In practice, the auxiliary classifier method has attracted the attention of many researchers interested in improving the convergence of losses and preventing the gradient from vanishing in deep neural networks [32]. Therefore, we assumed that this idea is also suitable for our problem since the auxiliary classifier can compensate for valuable information that is missing during the training process and optimize the network by aggregating pivotal features of the X-ray image in an intermediate layer.

To develop a deep learning network that can reliably diagnose maxillary sinusitis from X-ray images, we first developed three different models by combining basic convolutional modules, dense modules, and inception modules to form MVNet. Then, each model was reproduced in six variations depending on whether the auxiliary classifier is embedded or not. The models to which the auxiliary classifier method was applied had higher performance than the models to which the auxiliary classifier method was not applied. A significant difference was found for Aux-MVNet, while the performance for the other models increased slightly. Although only Aux-MVNet showed a significant performance increase, we conclude that these encouraging results have led to the auxiliary approach being able to optimize the low-level feature map which plays a vital role in the diagnosis of PNS using uncleaned radiographs.

It is also worth mentioning that our proposed system is equipped with an end-to-end detection network to automatically recognize maxillary regions in Waters’ view and Caldwell’s view. Thanks to the introduction of the detection network, we were able to evaluate the classification performance by dividing it into left and right maxillary sinuses. Conventional studies [13] dealing with classification problems in maxillary sinusitis usually require manual segmentation of the region of interest (ROI) prior to commencing model training. Reliable automated ROI segmentation is definitely beneficial as it can bypass this tedious work. 

To classify sinusitis into left and right, we took advantage of RetinaNet which can perform ROI detection and multinomial classification in one step. The results of detecting the left and right maxillary sinuses using RetinaNet showed APs of 0.960 and 0.970 in Waters’ view. In Caldwell’s view, the results of left and right maxillary sinus detection were excellent with APs of 0.882 and 0.872, respectively. The detection of the maxillary sinus region is not a challenging task, but we demonstrated that the RPN-based detection of the left and right maxillary sinus regions is reliable.

We performed the statistical analysis of network evaluation separately for the left and right maxillary sinuses. The Aux-MVNet achieved an AUC of 0.750 in the left maxillary sinus and an AUC of 0.700 in the right maxillary sinus. However, our study suffered from the lack of study data and data imbalance problems. In fact, the accuracy for sinusitis level-1, especially, for sinusitis level-1 in the right sinus was significantly lower than that of other classes (accuracy < 0.600). Therefore, the proposed approach in all settings is expected to achieve better classification performance for distinguishing sinusitis level-1 from other classes.

## 5. Conclusions

We proposed an Aux-MVNet to aid the diagnosis of maxillary sinusitis using Waters’ and Caldwell PNS radiographs. The auxiliary classifier in Aux-MVNet has significantly improved the classification performance of our model. We also demonstrated that our cascaded network can provide the estimated region of maxillary sinus and sinusitis levels. PNS radiographs are still primarily used in the ENT departments to diagnose sinusitis, so we expect that our network can support clinical decisions in maxillary sinusitis. In future studies, we plan to improve the clinical decision-making ability of our network by comparing the proposed method with otolaryngologists.

## Figures and Tables

**Table 1:** Network architectures used to compare sinusitis classification performance.

Basic MVNet	MVNet with Dense Module	MVNet with Inception-v1 Module
Input shape: (N, 512, 512, 1)		
Conv5, 16		
Maxpool2, stride2		
Conv5, 32	6 × dense block Transition layer	Inception-v1 block
Maxpool2, stride2		
Conv3, 64	12 × dense block Transition layer	Inception-v1 block
Drop 0.7, $GAP_{Aux}$ *		
Maxpool2, stride2		
Conv3, 128	24 × dense block Transition layer	Inception-v1 block
Maxpool2, stride2		
Conv3, 256	16 × dense block Transition layer	Inception-v1 block
Drop 0.3 *		
${\mathrm{GAP}}_{main}$		
Fully connected layer with sigmoid		
Output shape: (N, 6)		

Abbreviations: Conv5, 5 × 5 convolution filter; Maxpool2; 2 × 2 maxpool filter; Conv1, 1 × 1 convolution filter; Conv3, 3 × 3 convolution filter; Drop, drop out; GAP, global average pool.

**Table 2:** Average AUC metrics for each model are provided, and *p*-values are computed by comparing the ROC of each model to the MVNet model with an auxiliary classifier, where we used the statistical significance level of 0.05. The networks with the best performance in each region of sinusitis are indicated in bold.

	Area Under the Curve			*p* Value *
	Left	Right	Total	
MVNet	0.552	0.637	0.602	<0.001
MVNet with auxiliary classifier	**0.750**	0.700	**0.722**	-
Dense MVNet	0.695	0.723	0.706	=0.001
Dense MVNet with auxiliary classifier	0.703	0.722	0.709	=0.005
Inception MVNet	0.583	0.671	0.621	<0.001
Inception MVNet with auxiliary classifier	0.678	**0.753**	0.710	=0.002

* The *p* values for comparison of ROC. The ROC comparison analysis was performed in total region of sinus, which combined all classifications of left and right sinusitis. Abbreviations: MVNet, multi-view network; AUC, area under the curve.

**Table 3:** The evaluation result of Aux-MVNet.

	Normal Sinus	Sinusitis Level 1	Sinusitis Level 2
**Left Sinus**			
Accuracy	0.676	0.687	0.704
Sensitivity	0.796	0.536	0.700
Specificity	0.536	0.809	0.705
**Right Sinus**			
Accuracy	0.692	0.559	0.712
Sensitivity	0.697	0.622	0.679
Specificity	0.689	0.552	0.734
**Total**			
Accuracy	0.681	0.571	0.724
Sensitivity	0.708	0.656	0.625
Specificity	0.647	0.536	0.770

## Data Availability

The datasets generated and/or analyzed during the current study are not publicly available because permission to share patient data was not granted by the institutional review board, but they are available from the corresponding author upon reasonable request.
